# Cisplatin-based chemotherapy with or without bevacizumab for Chinese postmenopausal women with advanced cervical cancer: a retrospective observational study

**DOI:** 10.1186/s12885-020-06854-w

**Published:** 2020-05-05

**Authors:** Xiaoli He, Jun Liu, Li Xiao, Mingdong Zhao, Tingting Su, Tiejian Liu, Guowei Han, Yue Wang

**Affiliations:** 1 Department of Gynaecology, Henan Provincial People’s Hospital, People’s Hospital of Zhengzhou University, People’s Hospital of Henan University, No. 7, Weiwu Road, Jinshui District, Zhengzhou, 450000 China; 2 Department of Hepatobiliary Surgery, The First Affiliated Hospital, Sun Yat-sen University, No. 58, Zhongshan 2nd Road, Yuexiu District, Guangzhou, 510080 China; 3 Department of Gynaecology and Obstetrics, The Central Hospital of Wuhan, Tongji Medical College, Huazhong University of Science and Technology, No. 26, Shengli Street, Jiang’an District, Wuhan, 430014 Hubei China; 4 Department of Orthopaedics, Jinshan Hospital, Fudan University, Longhang Road No. 1508, Jinshan District, Shanghai, 201508 China; 5 Department of Gynaecology, The Obstetrics and Gynecology Hospital of Fudan University, No. 419, Fangxie Road, Huangpu District, Shanghai, 200011 China; 6 Department of Neurosurgery, The Third Affiliated Hospital of Southern Medical University, No.183, Zhongshan avenue west, Tianhe District, Guangzhou, 510630 China; 7 Department of Orthopaedics, The First Affiliated Hospital, Sun Yat-sen University, No. 58, Zhongshan 2nd Road, Yuexiu District, Guangzhou, 510080 China

**Keywords:** Bevacizumab, Cervical cancer, Chemotherapy, Overall survival, Progression-free survival

## Abstract

**Background:**

The purpose of this study was to assess the efficacy and safety of cisplatin-based chemotherapy with or without bevacizumab (BEV) in Chinese women with advanced cervical cancer (ACC).

**Methods:**

For this observational study, we analysed the data of 316 Chinese women with ACC who were treated at the Henan provincial people’s hospital between Jan 1, 2014, and Dec 31, 2018, with cisplatin-based chemotherapy plus BEV (CB) or cisplatin-based chemotherapy alone (CA) until disease progression, unacceptable toxicity, or death. The co-primary endpoints were overall survival (OS) and progression-free survival (PFS); the secondary endpoint was the occurrence of adverse events (AEs).

**Results:**

A total of 264 patients with ACC were included in the assessment (CB, *n* = 130 and CA, *n* = 134). At a median follow-up of 38 months (IQR 36–40), the median OS in the CB cohort was significantly longer than that in the CA cohort (hazard ratio [HR] 1.21, 95% confidence interval[CI] 1.14–1.73; *p* = 0.002); additionally, the median PFS was 345 days (95% CI, 318–372) for CB and 261 days (95% CI, 165–357) for CA(HR 1.61, 95% CI 1.12–2.17; *p* = 0.000). Significant differences were noted between groups in terms of thrombosis/embolism, neutropenia, and febrile neutropenia.

**Conclusions:**

In Chinese women with ACC, cisplatin-based chemotherapy plus BEV is associated with improved survival compared to cisplatin-based chemotherapy alone. This finding suggests a positive survival benefit of anti-angiogenesis therapy in this population.

## Background

Advanced cervical cancer (ACC) is usually regarded as a devastating disease affecting women worldwide because of its association with increased morbidity and mortality [1–6]. The incidence of ACC and associated mortality in China have been increasing since 2005 [7]. Treatment of ACC continues to be a challenge, although early-stage cancers may potentially be cured with radical surgery [7–10]. Five-year survival rates for patients with ACC varied from 4 to 15% within different study cohorts [3, 11]. Although the clinical outcomes for such cases have improved, the optimal strategy for ACC is still debatable [2, 8, 10, 12].

For patients with ACC, cisplatin-based chemotherapy has been regarded as the standard processing scheme, as in previous reports [13–17]. Nevertheless, findings from the Gynecologic Oncology Group (GOG)-240 randomized phase III trial [18] indicated that the incorporation of bevacizumab (BEV) with chemotherapy for recurrent, persistent or metastatic cervical cancer markedly increased the survival benefit. A recent phase III trial [19] using a 2 × 2 factorial design that was conducted to verify whether chemotherapy with or without BEV improves overall survival (OS) in women with ACC showed proof-of-concept of the efficacy and tolerability of anti-angiogenesis therapy in ACC because the sustained benefit conferred by chemotherapy plus BEV was evidenced by separated survival curves. Furthermore, the prospective validation of pooled prognostic factors in patients with ACC treated with chemotherapy with or without BEV demonstrated that the benefit to undergoing BEV tended to be remarkable in the moderate- and high-risk subgroups [19, 20]. Several studies [11, 18, 21] have tried to assess whether BEV is independently associated with survival. Nevertheless, such studies have had small sample sizes, have included patients undergoing drug treatment without the stratification of outcomes, or failed to adjust for some approved confounders. Furthermore, data regarding Chinese women with ACC who were treated with BEV-containing chemotherapy are extremely limited.

With these limitations in mind, we aimed to confirm whether Chinese women with ACC who are undergoing cisplatin-based chemotherapy plus BEV had greater survival benefits than those receiving cisplatin-based chemotherapy alone from Henan provincial people’s hospital from 2014 to 2018.

## Methods

### Study design and patient eligibility

Data for 350 postmenopausal women with ACC were identified and retrieved from the Henan Provincial People’s Hospital from Jan 1, 2014, to Dec 31, 2018. All demographic, clinicopathological, treatment, and survival data were obtained by trained clinical reviewers from the medical charts and telephone interviews with patients. The main inclusion criteria were as follows: postmenopausal women without menstruation for 12 consecutive months [13]; aged 55–75 years; metastatic, persistent, and recurrent cervical carcinoma; a history of papillomavirus (HPV) infections; at least one measurable lesion assessed according to the Response Evaluation Criteria in Solid Tumours (RECIST) version 1.1; adequate haematological levels, hepatic function, bone marrow function and renal function, as reported [19, 20, 22]; a GOG performance status score of 0 (fully active) or 1 [18]. The main exclusion criteria were as follows: patients with severe organ failure; rectofistula and/or vesical fistula; uncontrolled metabolic dysfunction; deaths from treatment-independent hypertension, cardiovascular events, and pneumonia; non-healing wounds; tumours invading major blood vessels; a high risk of bleeding; a thromboembolism event; cerebrovascular accident or coma lasting more than 24 h; delirious or otherwise cognitively impaired [23]; no or poor pretreatment image data; or inadequate medical records. The co-primary endpoints were OS and PFS; the secondary endpoint was the incidence of AEs.

### Study design and treatment

A retrospective, single-centre study was conducted in which eligible patients received chemotherapy with or without BEV [18]. The intravenous chemotherapy regimen consisted of cisplatin (at a dose of 50 mg per square metre of body surface area) plus paclitaxel (at a dose of 175 mg/m2 on day 1); the intravenous BEV regimen was a dose of 15 mg/kg on day 1; the regimens for chemotherapy plus BEV (CB) and chemotherapy alone (CA) were repeated at 21-day intervals, as described [18]. Treatment was performed until disease progression, withdrawal, unacceptable AEs, death, or if the patient had a complete response.

### Definitions of the descriptive variables

ACC was defined as metastatic, persistent, or recurrent cervical carcinoma, which was confirmed by a central pathology laboratory and through clinical imaging evidence. The co-primary endpoint measures were survival curves for OS and PFS. OS was computed from the date of drug treatment to the date of either death from any cause or final follow-up; PFS was calculated from the date of drug treatment to the date of either progression or death from any cause. Progression was defined according to the modified RECIST criteria. Disease assessment by contrast-enhanced computed tomography (CT) or magnetic resonance imaging (MRI) was performed at least every other month, regardless of patients exhibiting disease progression or not. Details related to disease assessment and tumour measurements were assessed according to the RECIST guidelines version 1.1 [24]. Safety assessment, which was performed according to the US national cancer institute’s patient-reported outcomes version of the common terminology criteria for adverse events (PRO-CTCAE) [25] during each cycle, was performed at least every 2 weeks for the initial 12 weeks and repeated at least every 4 weeks until disease progression, withdrawal, unacceptable AEs, death, or if the patient had a complete response. BEV could be delayed or discontinued based on the occurrence, duration, and severity of AEs.

### Statistical analysis

Baseline variables, treatment histories, and dates of first administration of drugs were obtained, along with dates of progression, final follow-up, and survival. We used Fisher’s exact or χ^2^ tests for categorical variables and the Mann-Whitney U test for continuous variables. Survival analysis was estimated with the Kaplan-Meier approach, regardless of the duration or type of therapy received. The secondary endpoint measure was occurrence of AEs. Patients with missing data were excluded from the final analysis. The median follow-up was calculated by the reverse Kaplan-Meier approach. Hazard ratios (HRs) were estimated using a Cox proportional hazard model with a 95% Wald confidence interval (95% CI) [18]. All *p* values were two-sided with the level of significance set at < 0.05. We performed data management and analyses with SPSS version 24.0 (IBM, Inc., NY, USA).

## Results

### Baseline characteristics

**Fig. 1:**
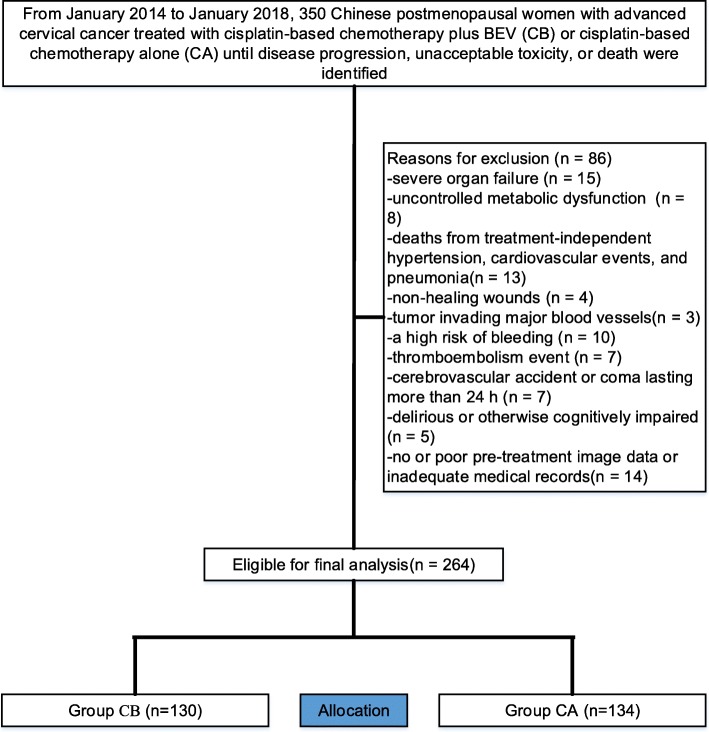
Flow diagram demonstrating the methods used to identify studies to retrospectively evaluate the efficacy and safety of cisplatin-based chemotherapy with or without bevacizumab (BEV) in Chinese women with advanced cervical cancer (ACC)

We analysed retrospective data from 350 patients with ACC, of whom 264 patients (CB, *n* = 130, mean age 67.22 years [SD 5.33] and CA, *n* = 134, 67.43 years [SD 7.53]) were identified for study eligibility (Fig. 1). Baseline characteristics, which were well balanced between both cohorts, are summarized in Table 1. The median follow-up for both cohorts was 38 months (IQR 36–40). The median number of cycles was 9 (range, 1–34) for CB and 10 (range, 1–39) for CA. Two hundred and twenty-eight(86%) patients discontinued the intervention, mainly attributed to disease progression (45% for CB vs. 33% for CA) and adverse events(21% for CB vs. 13% for CA). At final follow-up, sixteen patients that initially had tumour infiltration developed rectofistula and/or vesical fistula(5[3.8%] for CB vs. 11[8.2%] for CA, *p* = 0.137).

**Table 1:** Patient demographics between groups

Variable	CB (*n* = 130)	CA (*n* = 134)	*p*-value
Age at onset (years)	67.22 ± 5.33	67.43 ± 7.53	0.346^*a*^
Histology, n (%)			0.885^*b*^
Squamous	74(57)	76(57)	
Adenocarcinoma	43(33)	48(36)	
Other	13(10)	10(7)	
Disease presentation, n (%)			0.501^*b*^
Recurrent	35(27)	33(25)	
Persistent	27(21)	25(19)	
Stage IVB	68(52)	76(56)	
Prior treatment, n (%)			0.489^*b*^
Radical surgery	14(11)	22(16)	
Radical radiotherapy	25(19)	27(20)	
Radiotherapy adjuvant	34(26)	30(22)	
Radical chemoradiotherapy	19(15)	16(12)	
Palliative radiotherapy	27(21)	23(17)	
No prior treatment	11(8)	16(13)	
Duration of treatment (mo)	28.63 ± 7.15	28.35 ± 7.42	0.225^*a*^
Performance status (ECOG), n (%)			0.955^*b*^
0	47(36)	48(36)	
1	83(64)	86(64)	
GOG performance status, n (%)			0.970^*b*^
0	42(32)	43(32)	
1	88(68)	91(68)	
Number of metastatic sites, n (%)			0.234^*b*^
3	33(25)	27(20)	
> 3	77(59)	81(60)	
unknown	20(16)	26(20)	
Prior pelvic radiotherapy, n (%)			0.889^*b*^
Yes	60(46)	63(47)	
No	70(54)	71(53)	

^*a*^*Analysed using an Independent-Samples t-test;*^*b*^*Analysed using the Mann-Whitney U test. CB* cisplatin-based chemotherapy plus bevacizumab*, CA* cisplatin-based chemotherapy alone*, GOG* Gynecologic Oncology Group*, ECOG* Eastern Collaborative Oncology Group

### Comparison of efficacy

**Fig. 2:**
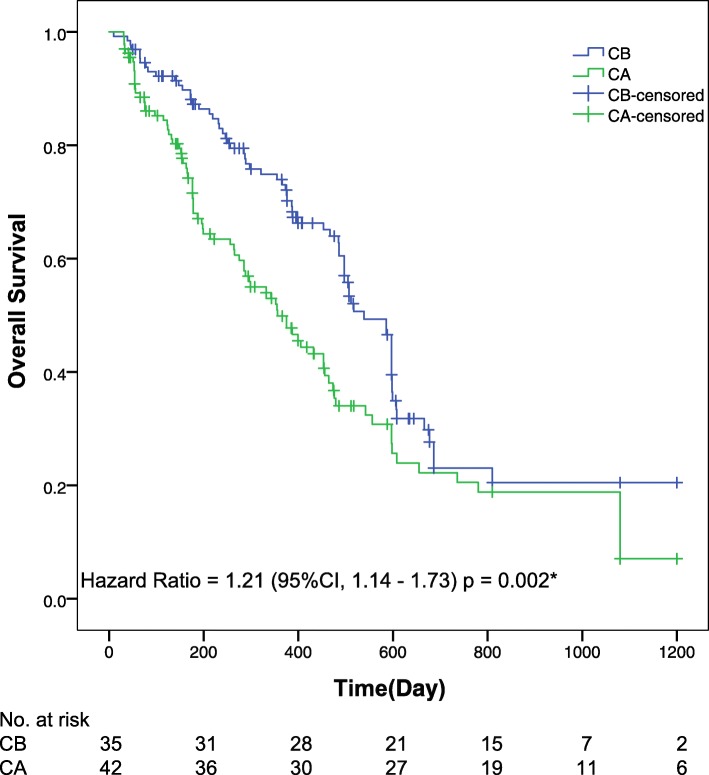
**Kaplan**-**Meier curves for overall survival.** The median overall survival was 18.0 months (95% confidence interval [CI], 16.1–19.9) and 11.9 months (95% CI, 8.8–15.0) for the CB and CA groups, respectively. Significant differences were detected in overall survival between groups. *The hazard ratio was calculated using a Cox proportional hazards model, with the age, the site of primary tumour, the number of metastatic sites, and the performance status used as covariates and CB/CA therapy as the time-dependent factor. With respect to the overall survival, the results of a log-rank test reported *p* = 0.002

**Fig. 3:**
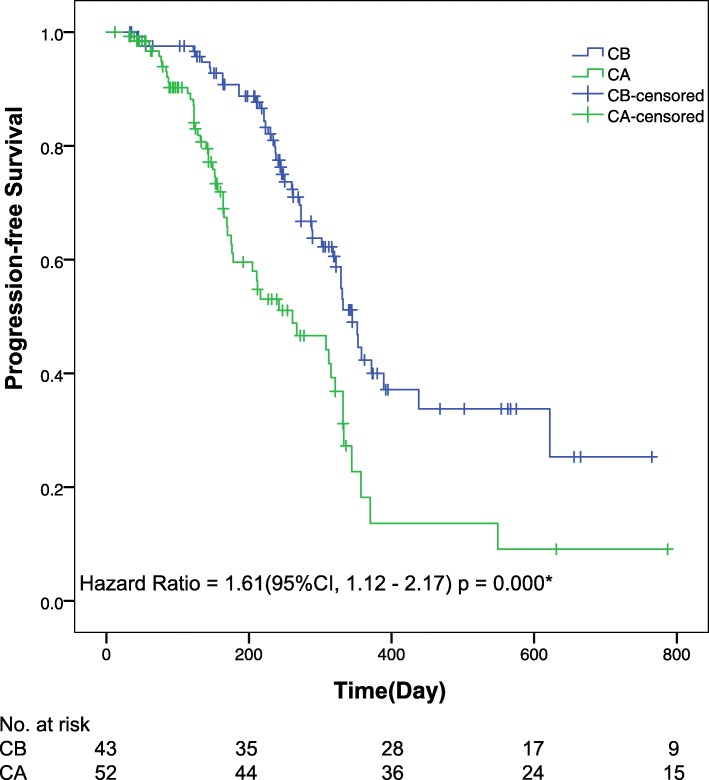
**Kaplan**-**Meier curves for progression-free survival.** The median progression-free survival was 11.5 months (95% confidence interval [CI], 10.6–12.4) and 8.7 months (95% CI, 5.5–11.9) for the CB and CA groups, respectively. Statistically significant differences were observed in progression-free survival between groups. *The hazard ratio was calculated using a Cox proportional hazards model, with age, the site of primary tumour, the number of metastatic sites, and the performance status used as covariates and CB/CA therapy as the time-dependent factor. With respect to progression-free survival, the results of a log-rank test reported *p* = 0.000

Final analysis of patient response showed that approximately 56% of patients responded on cisplatin-based chemotherapy with and without BEV. For the CB-treated cohort, the median OS was reached (95% CI 18.0 months to not reached); the 1-year OS has not been reached; the 2-year OS was 45% (41–52). For the CA-treated cohort, the median OS was also reached (95% CI 11.9 months to not reached); the 1-year OS has not been reached; the 2-year OS was 38% (34–42). At final follow-up, the median OS was 540 days (95% CI, 483–597) in the CB group and 357 days (95% CI, 264–450) in the CA group; the median PFS was 345 days (95% CI, 318–372) in the CB group and 261 days (95% CI, 165–357) in the CA group. Significant differences were observed between groups in both the median OS (HR 1.21, 95% CI 1.14–1.73; *p* = 0.002) (Fig. 2) and median PFS (HR 1.61, 95% CI 1.12–2.17; *p* = 0.000) (Fig. 3).

### Comparison of safety

The main drug-related AEs, regardless of the study drug relationship, are summarized in Table 2. CA tends to be safer when patients have neutropenia based on the toxicity profile compared to that of CB. The frequency and severity of drug-related AEs were in line with the acknowledged safety profile. Grade 3 or higher thromboembolism was more frequent in the CB group than in the CA group (13.1% vs 5.2%; *p* = 0.026). Neutropenia or febrile neutropenia occurred in 35 cases (13.3%) among both groups, of which 9 occurred during the first 4 months in the CA group and 26 occurred during the first 7 months in the CB group. Additionally, AEs leading to dose interruptions or permanent discontinuation occurred in 37 cases (24 [18.5%] for CB-treated cohort and 13 [9.7%] for CA-treated cohort; *p* = 0.040), primarily owing to grade ≥ 3 neutropenia or thrombosis/embolism. Other grade 2 or higher AEs were fistula (occurring in 30 cases [11.4%]), hypertension (in 32 cases [12.1%]), bleeding (in 17 cases [6.4%]), proteinuria (in 26 cases [9.8%]), and pain (in 53 cases [20.1%]), which were not significantly different between groups. The incidence of other AEs of special interest (i.e., headache, emesis) was generally similar between the groups.

**Table 2:** Comparison of the incidence of main drug-related AEs of grade ≥ 2 between the groups at final follow-up

AEs	CB (*n* = 130)	CA (*n* = 134)	*p*-value
Thrombosis/embolism, grade ≥ 3, n (%)	17(13.1)	7(5.2)	0.026*^a^
Neutropenia, grade ≥ 4, n (%)	10(7.7)	3(2.2)	0.041*^a^
Febrile neutropenia, grade ≥ 3, n (%)	16(12.3)	6(4.5)	0.021*^a^
Fistula, grade ≥ 2, n (%)	8(6.2)	9(6.7)	0.852^a^
Fistula, grade ≥ 3, n (%)	7(5.4)	6(4.5)	0.733^a^
Hypertension, grade ≥ 2, n (%)	15(11.5)	17(12.7)	0.775^a^
Bleeding, grade ≥ 3, n (%)	9(6.9)	8(6.0)	0.752^a^
Proteinuria, grade ≥ 3, n (%)	11(8.5)	15(11.2)	0.456^a^
Pain, grade ≥ 2, n (%)	25(19.2)	28(20.9)	0.736^a^

^*^Statistically significant values.^*a*^*Analysed using the chi-square test. AEs* adverse events*, CB* cisplatin-based chemotherapy plus bevacizumab*, CA* cisplatin-based chemotherapy alone*, AEs* adverse events

## Discussion

To the best of our knowledge, this study is the largest so far on postmenopausal Chinese women with ACC who were treated with cisplatin-based chemotherapy with or without BEV. Our study met its co-primary endpoints; the BEV-containing regimen was associated with an increased survival benefit. The superiority of CB over CA in this setting tended to be positive. BEV-related AEs were similar to those observed in previous reports.

Several limitations should be considered. First, the retrospective nature of our analysis with this methodology decreased the power to draw reliable conclusions, and some potential variables (such as some medical diseases) could not be addressed in our analysis. Second, the relatively small sample size in the present study may have introduced bias. Third, generalizability was lacking owing to the study population involving only Chinese postmenopausal patients with ACC. Fourth, power might be underestimated, primarily due to our analysis involving repeated observations of each subject.

Our analysis determined survival with a longer follow-up and was consistent with previous findings [11, 13, 18] that CB improves survival benefit in patients with ACC, since the 3-year OS reported here (41%) is similar to that reported in a randomised, controlled, open-label, phase 3 trial (39%) [19]. In view of multiple regimens with noteworthy activity in ACC treatment, clinical OS results might be confounded by the availability of these regimens [18]. BEV, a humanized anti-VEGF monoclonal antibody, has already demonstrated remarkable activity in ACC, as assessed by response rate [11, 19]; however, the effect of BEV on survival benefit needs to be determined as an indication of definitive survival benefit [13]. Survival benefit has conventionally been considered the most dependable endpoint in assessing cancer-related treatments [18, 20, 21].

In a phase III randomized trial [19] using a 2 × 2 factorial design, 452 ACC patients from 164 institutions in the United States and Spain were enrolled and randomized to receive CB or CA and showed significant improvement in OS: 16.8 vs 13.3 months for the CB and CA groups, respectively (HR, 0.77; 95% CI, 0.62–0.95; *p* = 0.0068), and PFS also favoured BEV (HR 0.68; 95% CI 0.56–0.84; *p* = 0.0002). Additionally, a recent retrospective study [11] demonstrated a survival benefit of BEV when combined with chemotherapy in patients with recurrent, persistent or advanced cervical cancer. Why these analogous treatment regimens translated into corresponding gains in survival benefit is not confounding. In the current study, the large effect of CB on the treatment of ACC in the first 1 year with little effect thereafter was interesting.

Although chemotherapy plus BEV has been confirmed in patients with ACC in previous trials, data in the patient population remain limited [18, 20]. Recently, a randomized trial by Penson [21] assigned 390 evaluable ACC patients to analyse patient reported outcomes in GOG 240 and showed that CB significantly improves OS, PFS, and response rates compared to CA. In the ACC setting, it is important to evaluate any lengthening in the duration of PFS and OS. Nevertheless, frequent debate often occurs regarding the influence of the oestrogen, predominantly in the postmenopausal cohort [26–28]. To reduce the impact of oestrogen on survival in the present study, the primary strategy was to only include a postmenopausal cohort. For these individuals who were ineligible for radical resection but still have their disease confined to the uterus, uterus-directed therapies may play a prominent role in reducing tumour burden and increasing survival [28]. However, for patients with ACC, chemotherapy plus BEV might be a good choice that has exhibited a positive impact on survival [19, 21].

There remains a paucity of survival data in the previous trials of postmenopausal patients with ACC [11, 13, 18]. Although it recognizes a distinct separation of PFS and OS curves, favouring the continuation of BEV, the continuation of BEV beyond progression failed to produce promising outcomes [18]. Moreover, the absolute improvements in the survival benefit appear small for this postmenopausal cohort with ACC and tended to be in association with the timing of tumour assessments [29, 30]. In contrast to previous studies [18, 19, 21], nevertheless, further analysis showed no considerable interaction between the continuation of BEV beyond progression and survival benefit.

## Conclusion

The results reported here support the growing body of evidence that cisplatin-based chemotherapy plus BEV conferred a significant survival benefit versus cisplatin-based chemotherapy alone for Chinese women with ACC. In light of this finding, we are currently advocating for the incorporation of BEV with cisplatin-based chemotherapy as a clinical decision in this patient population. Future trials regarding the efficacy and safety of cisplatin-based chemotherapy plus BEV in a similar setting are needed.

## Data Availability

The datasets used and/or analysed during the current study are available from the corresponding author on reasonable request.

## Abbreviations

BEV: Bevacizumab
ACC: Advanced cervical cancer
CB: Cisplatin-based chemotherapy plus BEV
CA: Cisplatin-based chemotherapy alone
OS: Overall survival
PFS: Progression-free survival
AEs: Adverse events
HR: Hazard ratio
CI: Confidence interval
GOG: Gynecologic Oncology Group
RECIST: Response Evaluation Criteria in Solid Tumours
CT: Computed tomography
MRI: Magnetic resonance imaging
PRO-CTCAE: Patient-reported outcomes version of the common terminology criteria for adverse events
IQR: Interquartile range
SD: Standard deviation
